# PREOPERATIVE HOSPITALIZATION AS A BRIDGING STRATEGY FOR WEIGHT LOSS IN PATIENTS WITH BODY MASS INDEX = 50 KG/M^2^ WHO ARE CANDIDATES FOR BARIATRIC SURGERY

**DOI:** 10.1590/0102-6720202400058e1852

**Published:** 2025-01-20

**Authors:** Renata Ramos Severo, Fernando Santa-Cruz, Flávio Kreimer, André Bezerra de Sena, Álvaro Antônio Bandeira Ferraz

**Affiliations:** 1 Universidade Federal de Pernambuco, Postgraduate in Surgery – Recife (PE), Brazil; 2 Hospital dos Servidores do Estado, General Surgery Service – Recife (PE), Brazil; 3 Universidade Federal de Pernambuco, Hospital das Clínicas, General Surgery Service, Recife (PE), Brazil.

**Keywords:** Obesity, morbid, Bariatric surgery, Caloric restriction, Weight loss, Obesidade mórbida, Cirurgia bariátrica, Restrição calórica, Redução de peso

## Abstract

**BACKGROUND:**

Preoperative hospitalization with the purpose to obtain more effective weight loss provides intensive care for patients who have a higher body mass index (BMI) and associated diseases that involve a greater risk of peri- and postoperative complications. It is a therapeutic strategy that can make it possible to overcome obstacles related to the difficulty of adhering to obesity treatment.

**AIMS:**

To analyze the implementation of a preoperative hospitalization strategy for weight loss in patients eligible for bariatric surgery.

**METHODS:**

Retrospective study that included 194 patients with a BMI=50 kg/m^2^. They were grouped according to preoperative preparation strategies: inpatient (n=32) and outpatient (n=162), who underwent Roux-en-Y gastric bypass (RYGB) or sleeve gastrectomy (SG) between 2010 and 2020. The groups were compared regarding preoperative weight loss before and after the strategies and postoperative up to two years after surgery.

**RESULTS:**

Most patients were female and there were significant differences in age group (an average of 42.94 years in the preoperative hospitalization strategy group and 37.73 in the outpatient strategy group). The mean BMI in the hospitalized group was 63.01±8.72 kg/m^2^, and in the outpatient group it was 54.95±4.31 kg/m^2^. There was a significant difference only between initial and preoperative weight in the hospitalized group. Furthermore, the difference between initial weight and last recorded weight up to two years after surgery was significant in each group. The occurrence of associated diseases was higher in the outpatient group.

**CONCLUSIONS:**

Patients following the preoperative hospitalization strategy experienced significant weight loss before surgery.

## RESUMO

**RACIONAL:**

A internação pré-operatória com o propósito de obter uma perda de peso mais efetiva, proporciona cuidados intensivos para pacientes que apresentam IMC mais alto, e doenças associadas que envolvem maior risco de complicações peri e pós-operatórias. É uma estratégia terapêutica que pode possibilitar superar obstáculos relacionados à dificuldade de adesão ao tratamento da obesidade.

**OBJETIVOS:**

Analisar a implementação de uma estratégia de hospitalização pré-operatória para perda de peso em pacientes elegíveis para cirurgia bariátrica.

**MÉTODOS:**

Estudo retrospectivo que incluiu 194 pacientes com índice de massa corpórea (IMC)=50 kg/m^2^. Os pacientes foram agrupados de acordo com a estratégia de preparo pré-operatório: internamento (n=32) e ambulatorial (n=162), que foram submetidos ao bypass gástrico em Y de Roux (BGYR) ou gastrectomia vertical (GV), entre 2010 e 2020. Os grupos foram comparados de acordo com a perda de peso pré-operatória decorrentes das estratégias mencionadas, e com a perda de peso pós-operatória após um seguimento de dois anos da cirurgia.

**RESULTADOS:**

A maioria dos pacientes foi do sexo feminino e houve diferença significativa em relação à idade entre os grupos (42,94 anos no grupo hospitalização pré-operatória e 37.73 anos no grupo ambulatorial). O IMC médio no grupo hospitalização foi 63.01±8.72 kg/m^2^, e no grupo ambulatorial foi 54.95±4.31 kg/m^2^. Houve diferença significativa apenas entre o peso inicial e o pré-operatório final imediatamente antes da cirurgia (após a estratégia) no grupo hospitalização. Ainda, a diferença entre o peso inicial e o peso pós-operatório final foi significante em ambos os grupos. A prevalência de doenças associadas foi maior no grupo ambulatorial.

**CONCLUSÕES:**

Pacientes com IMC=50 kg/m^2^ submetidos à estratégia de hospitalização pré-operatória apresentaram perda de peso significativo antes da cirurgia bariátrica.

## INTRODUCTION

Bariatric surgery is recognized as the main and most effective treatment approach for the population with a body mass index (BMI) equal to or greater than 50 kg/m^2^. However, to ensure a safe procedure, it is recommended to reduce the BMI preoperatively, resulting in a weight loss of between 10 and 15% of the initial weight^19^. Furthermore, essential measures include stopping smoking and alcohol consumption, glycemic control, maintaining blood pressure within adequate parameters, implementing a dietary program, incorporating regular physical activity, and monitoring by a multi-professional team, including medication use, among other recommendations^8,9,12^.

The literature lists the following as effective preoperative strategies for bariatric surgery in patients with a BMI of 50 kg/m^2^ or more: intragastric balloon implantation, low-calorie diet (LCD), pharmacological therapy, multi-professional monitoring, and hospitalization. These strategies are usually combined^8^. However, they are not always available in public health services.

Preoperative hospitalization with the purpose to obtain more effective weight loss provides intensive care for patients who have a higher BMI and associated diseases that involve a greater risk of peri- and postoperative complications. This preoperative hospitalization is a therapeutic strategy that can make it possible to overcome obstacles related to the difficulty of adhering to obesity treatment and overcome issues associated with objective conditions for its effectiveness. Examples include ensuring adequate transportation for patients to attend outpatient appointments, performing specialized exams due to the need to adapt technological infrastructure that is often incompatible with patients’ anthropometry and ergonomics, maintaining an LCD, vitamin supplementation, and drug treatment due to the high cost^11,16,17^.

Therefore, it is essential to study accessible strategies that enable preoperative weight loss in patients with a BMI=50 kg/m^2^ who are candidates for bariatric surgery. The hypothesis tested here is that hospitalization is feasible and effective regarding BMI reduction and loss of excess weight before bariatric surgery.

This study aimed to evaluate the outcome of two preoperative bridge strategies for bariatric surgery regarding weight loss in patients with BMI=50 kg/m^2^.

## METHODS

### Study design

This study is a prospective cohort that included a population of 194 patients with BMI=50 kg/m^2^ and at least 18 years old who underwent Roux-en-Y gastric bypass (RYGB) or sleeve gastrectomy (SG), from January 2010 to December 2020. According to the preoperative weight loss strategy, the population was divided into two groups: inpatient (32 patients) and outpatient (162 patients). Data was gathered prospectively through an electronic database. Patients who had undergone revisional surgery were excluded from the analysis.

### Data collection and group formation

The sample was non-probabilistic and selected through convenience. Data were recorded at four different moments: before the preoperative weight loss strategy, i.e., when the patient started the program; after the strategy (immediately before surgery); six months postoperative; and two years postoperative. For the inpatient group, the preoperative hospitalization length was between two and six months until the surgery was performed. Meanwhile, the outpatient follow-up for weight loss was 18 months and could be up to two years.

In both strategies, care was provided by a multi-professional team comprising a bariatric surgeon, a social worker, a nurse, a psychologist, a nutritionist, a speech therapist, and a physical education professional, with an average of two to six appointments for each specialty. In addition to monitoring by a multi-professional team, all patients were assessed and monitored by doctors: endocrinologists, cardiologists, pulmonologists, and others according to the needs of each case, with reports being issued for clearance to undergo the surgical procedure. Patients in each group were categorized according to sociodemographic, anthropometric, and clinical variables based on data collection. Anthropometric measurements included absolute weight, BMI, excess weight, and percentage loss of excess weight. Preoperative weight was defined as the patient's weight recorded in the medical records on the preanesthetic evaluation form when undergoing bariatric surgery.

In both strategies, patients underwent multi-professional monitoring and low-calorie diet (LCD). However, for the inpatient group, the individuals would undergo a LCD, which involves a deficit of 1,200 to 1,500 kcal per day, followed by a very low-calorie diet (VLCD) with an intake of 800 kcal per day if tolerated. These diets aim to achieve a preoperative weight loss between 15 and 20% during hospitalization^14^. Each case is adjusted according to its needs. The outpatient strategy proposes the same LCD, but patients’ adherence to the diet in the home environment is low and poorly controlled^15^. The detailed protocol is attached as supplementary file.

When comparing the two strategies, the results relating to absolute weight loss, BMI reduction, reduction in excess weight, and percentage loss of excess weight were analyzed. Therefore, we considered the condition of the patients before and after undergoing the strategies and the postoperative period up to two years after the procedure^1^.

All procedures performed in this study involving human participants were in accordance with the ethical standards of the institutional research committee and the 1964 Helsinki declaration and its later amendments. The study was approved by the Ethics Committee of the Institution (No. 19800819.0.0000.880).

### Statistical analysis

The data were analyzed descriptively using absolute and percentage frequencies for the categorical variables and the measures: mean, standard deviation (mean±SD) and median, and the 25^th^ and 75^th^ percentiles (median (P25; P75) for the numerical variables. We used Pearson's chi-square test or Fisher's exact test to compare the two groups regarding the categorical variables when the condition for using the chi-square test was not met, and Mc Nemar's test to compare the evaluations in each group. Regarding the numerical variables, the comparison between groups used the Student's t-test with equal variances or Mann-Whitney, and the comparison between evaluations used the paired t-test or Wilcoxon. We chose the Student's t-test, with equal variances, when the data in each group showed a normal distribution, and the Mann test if normality was rejected. We chose the paired Student's t-test when the difference between the evaluations showed a normal distribution and the paired Wilcoxon test if normality was rejected. Normality was verified using the Shapiro-Wilk test, and equality of variances was verified using Levene's F-test. The margin of error used for the statistical tests was 5%. The data was recorded in an Excel spreadsheet, and the program used for statistical calculations was IMB Statistical Package for the Social Sciences (SPSS), version 25.

## RESULTS

The sample comprised 194 patients with a BMI=50 kg/m^2^ who were submitted to a preoperative weight loss strategy before undergoing RYGB or SG. The groups were mainly formed by women (hospitalization: 65.6% x outpatient: 70.4%) with a mean age of 42.94±13.30 and 37.73±10.55, respectively (p=0.028). There were no differences between groups regarding drinking or smoking habits, high blood pressure, diabetes, dyslipidemia or sleep apnea. The group with the preoperative hospitalization strategy comprised a population with lower education levels, with the majority having completed only elementary school (46.4%), while most of the outpatient strategy group had completed high school (48.6%). The hospitalized group also had lower income, with 32% earning up to one minimum wage compared to 17.5% in the outpatient group (Table 1).

**Table 1:** Sample characterization.

Variable		Inpatient	Outpatient	Total group	p-value
Age: Mean±SD		42.94±13.30	37.73±10.55	38.59±11.18	p(1)=0.028*
Sex: n (%)					
	Male	11 (34.4)	48 (29.6)	59 (30.4)	p(2)=0.594
	Female	21 (65.6)	114 (70.4)	135 (69.6)	
Drinking habit n (%)					
	Yes	10 (31.3)	53 (34.2)	63 (33.7)	p(2)=0.566
	Former drinker	1 (3.1)	12 (7.7)	13 (7.0)	
Smoking habit n (%)					
	Smoker	6 (18.8)	21 (13.3)	27 (14.2)	p(2)=0.673
	Former smoker	5 (15.6)	31 (19.6)	36 (18.9)	
	HBP n (%)	24 (75.0)	118 (73.3)	142 (73.6)	p(2)=0.841
	T2D n (%)	9 (28.1)	24 (14.9)	33 (17.1)	p(2)=0.070
	Dyslipidemia n (%)	4 (12.5)	10 (6.2)	14 (7.3)	p(3)=0.256
	Sleep apnea n (%)	1 (3.1)	4 (2.5)	5 (2.6)	p(3)=1.000

SD: standard deviation; HBP: high blood pressure; T2D: type 2 diabetes.

* significant difference at the 5.0% level.

(1) Mann-Whitney test;

(2) Pearson's chi-squared test;

(3) Fisher's exact test.

The median initial weights and BMI were comparatively evaluated within each group, as well as the preoperative weight. The group undergoing a preoperative hospitalization strategy had significantly higher median weight and BMI compared to the outpatient strategy group (163.72 vs. 146.27 kg) and (63.01 vs. 54.95 kg/m^2^). There was a significant preoperative weight loss in the hospitalized group compared to the outpatient group (11.50±18.60 vs. 0.92±11.45 kg). There was a significant difference in the final postoperative weight loss, although the excess weight loss was similar between the two groups during the 2-year follow-up (59.45±33.60 kg vs. 47.68±17.63 kg) and (58.77±27.95% vs. 60.74±19.80%). The operative time differed between groups, being significantly higher in the outpatient group (224.8±47.3 x 191.0±74.9; p=0.02, p<0.05) (Table 2).

**Table 2:** Statistics on weight, excess weight, body mass index, weight loss, operation time and early postoperative complications according to the hospitalization and outpatient groups.

Variable		Inpatient	Outpatient	p-value
		Mean±SD	Mean±SD	
Initial weight (kg)		163.72±29.24	146.27±22.64	p(1)=0.001*
Preoperative weight (kg)		152.22±24.49	145.36±23.01	p(1)=0.101
Absolute diff. (initial x preoperative) (kg)		11.50±18.60	0.92±11.45	p(1)<0.001*
Absolute diff. (initial x last weight up to two years after surgery) (kg)		59.45±33.60	47.68±17.63	p(2)=0.016*
%EWL		58.77±27.95	60.74±19.80	p(1)=0.657
Initial BMI		63.01±8.72	54.95±4.31	p(1)<0.001*
Preoperative BMI		58.61±7.30	54.63±4.90	p(1)=0.003*
Postoperative weight				
	Six months	123.20±22.10	112.85±20.38	p(1)=0.029*
	Two years	98.52±21.70	91.61±19.38	p(2)=0.213
	Operative time (min)	191.0±74.9	224.8±47.3	p(3)=0.02*
Early complications				
	Gastric leak n(%)	0	1 (0.6%)	-
	Pulmonary embolism n(%)	1 (3.1%)	1 (0.6%)	p<0.001

SD: standard deviation; kg: kilogram; diff: difference; %EWL: percentage of excess weight loss; BMI: body mass index;

* significant difference at the 5.0% level.

(1) Mann-Whitney test;

(2) Student's t-test with equal variances;

(3) paired Student's t-test;

(4) paired Wilcoxon test.

## DISCUSSION

High BMI has emerged as the main indication criterion for bariatric surgery, which is considered the most effective method for treating extreme obesity due to significant weight loss, long-term maintenance of this loss, and control or remission of the main associated diseases^13^. However, for patients with a BMI of 50 kg/m^2^ or more, the response to surgery regarding weight loss is lower, and the incidence of postoperative complications is significantly higher^6,10^.

One of the main strategies for reducing risks in preoperative patients is weight loss, with a recommended reduction of 10 to 15% of the initial weight. It helps control associated comorbidities and reduces surgical risk. LCD's are one of the best strategies for this purpose^4^.

This study analyzes the implementation of a preoperative hospitalization strategy for weight loss in an obesity treatment center, seeking to assess its impact on patients’ weight evolution. Most patients in the hospitalization group had a BMI of 60 kg/m^2^ or more (65.5%). Meanwhile, in the outpatient group, most had a BMI between 50 kg/m^2^ and 59.9 kg/m^2^ (85.8%).

Individuals enrolled in the inpatient group achieved an average preoperative weight loss of approximately 7.02%, equivalent to around 4.4 BMI points. In contrast, the outpatient group did not show significant preoperative weight loss, which may be a reflex of the poor adherence to the LCD prescribed and the absence of close surveillance. Postoperative results were similar in both groups, revealing no significant benefits in final weight loss. These results contrast with the findings of other studies, such as the cohort carried by Huerta et al.^7^ They observed advantages associated with preoperative weight loss in patients with a BMI=50 kg/m^2^. These included greater weight loss in the late postoperative period, reduced operating time, reduced perioperative and postoperative complications, and a substantial reduction in patient morbidity and mortality throughout follow-up.

A study carried out by Santo et al. which included 20 patients with obesity showed a mean BMI of 66 kg/m^2^ in an inpatient program similar to the one carried out in the present study. The authors observed a weight loss of 10% in 7.7 weeks, 15.2% in 15 weeks, and 19.7% in 21.3 weeks of hospitalization^15^. By week 14 (63% of the average length of stay), 78.3% of patients had achieved the desired weight loss. At this point, the weight loss became statistically insignificant.

These differences regarding the final outcomes related to weight loss may be explained by the heterogeneity between the two studied groups, where the "inpatient group" had a significant higher proportion of patients with BMI=60 kg/m^2^, therefore being prone to a poorer response to surgery when compared with patients with lower BMI ranges.

Another preoperative strategy for weight loss involves using an intragastric balloon (IGB). Recent clinical trials have shown that the balloon can be effective for this purpose, resulting in an average BMI loss of approximately −2.8 kg/m^2^. However, this value is lower than the −4.4 kg/m^2^ observed in the inpatient group of our study. Furthermore, using IGB has inherent complications, which add to the surgical morbidity, including abdominal pain, vomiting, and sometimes the need for a new procedure early on for removal due to intolerance^3^.

The operational cost and patients’ difficult access to the tertiary health network represent additional obstacles to the routine implementation of more invasive strategies such as IGB. Analysis of the sociodemographic profile of the studied population reveals a significant fragility, evidenced by the low schooling and income levels in both groups. This situation suggests difficulties in both preoperative and postoperative monitoring, especially due to the need to adhere to specific diets and take vitamin supplements^11^. These challenges are minimized during hospitalization, where multidisciplinary preoperative monitoring occurs. This fact is corroborated by evidence of the absence of preoperative weight loss in the group that underwent outpatient follow-up.

Faced with the challenge of surgical management, which is the mainstay of treatment for obesity in patients with BMI >50 kg/m^2 5^, preoperative hospitalization is a strategy to be considered in the reality of public health in Brazil. It helps with preoperative weight loss and provides patients with a better clinical and metabolic condition, which can be fundamental for the response to surgery^16^. This strategy aligns with one of the fundamental principles of the Brazilian Unified Health System: equity. It aims to provide a comprehensive set of therapeutic approaches targeted especially at those who need it most, ensuring a fairer and more inclusive approach^2^. Another factor that should be highlighted in this group of patients is that bariatric surgery policies favor surgery only as a last resource. Most patients with a BMI equal to or greater than 50 kg/m^2^ could have undergone bariatric surgery when their BMI was lower^18^.

This study's limitations include its observational nature and all the implications of this design. A randomized design would have added huge value for the present analysis; however, ethically, it would be a tricky process to implement since there is a higher tendency in our center to indicate the inpatient strategy for those individuals presenting higher values of BMI and higher surgical risk.

The national and international literature lacks studies on preoperative management strategies for patients with a BMI of 50 kg/m² or more. However, our results indicate that management through preoperative hospitalization seems to provide significant results for preoperative weight loss and a more effective approach in populations with a high degree of obesity. Nonetheless, they confirm the need to improve preoperative strategies for the population with a BMI of 50 kg/m^2^ or more.

## CONCLUSIONS

After undergoing the implementation of the preoperative hospitalization strategy with a supervised diet, the patients showed a significant difference in absolute weight loss in the preoperative period. This approach has proven to be safe and effective in achieving this goal, especially among the population of patients with higher BMI ranges. Thus, it adds another strategic alternative for the preoperative management of these individuals. However, there was no significant difference in the patients’ final weight loss. Studies with a higher level of evidence still must determine the real influence of the preoperative hospitalization strategy on the response of patients with a BMI=50 kg/m^2^ to bariatric surgery.

Central Message Preoperative hospitalization with the purpose to obtain more effective weight loss provides intensive care for patients who have a higher body mass index (BMI) and associated diseases that involve a greater risk of peri- and postoperative complications. It is essential to study accessible strategies that enable preoperative weight loss in patients with a BMI=50 kg/m2 who are candidates for bariatric surgery. This preoperative hospitalization is a therapeutic strategy, in public hospitals, that can make it possible to overcome obstacles related to the difficulty of adhering to obesity treatment.

Perspectives After undergoing the implementation of the preoperative hospitalization strategy with a supervised diet, the patients showed a significant difference in absolute weight loss in the preoperative period. This approach has proven to be safe and effective in achieving this goal, especially among the population of patients with higher BMI ranges. Thus, it adds another strategic alternative for the preoperative management of these individuals. However, there was no significant difference in the patients’ final weight loss.
